# Supplementary material for: Overexpression of Tamarix hispida ThTrx5 Confers Salt Tolerance to Arabidopsis by Activating Stress Response Signals
Source: Int J Mol Sci. 2020 Feb 10;21(3):1165. doi: 10.3390/ijms21031165 (PMC7037472; doi:10.3390/ijms21031165)
Supplement: Supplementary file 1 [file ijms-21-01165-s001.pdf]

Supplementary data

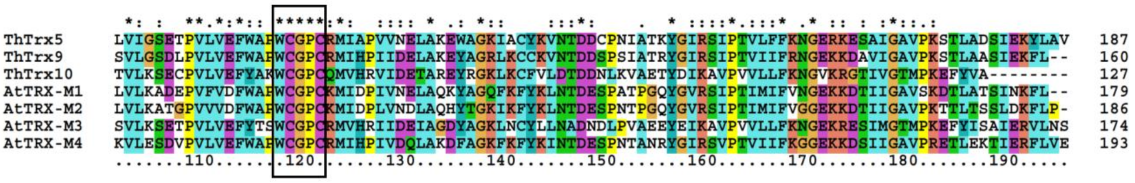

Supplementary Figure S1. Multiple Sequence Alignment of ThTrx Proteins of family I m Subclasses in Tamarix and Arabidopsis.The box highlights the WCGPC domains.

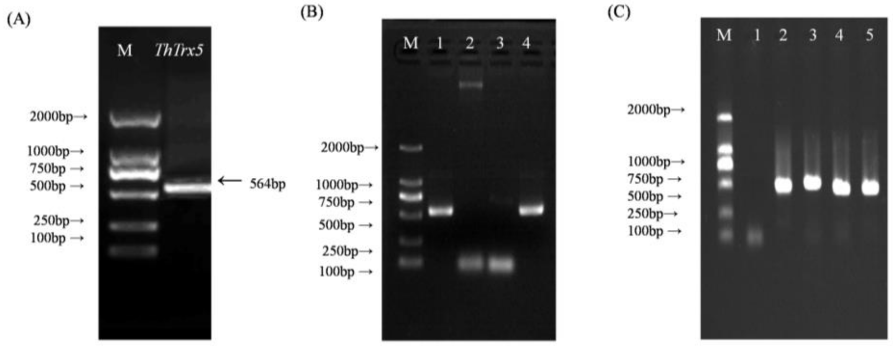

Supplementary Figure S2: *ThTrx5* gene cloning and plant expression vector construction process. (**A**) PCR amplified fragment of *ThTrx5* gene; (**B**) PCR detection of *ThTrx5* gene topo reaction M. DL2000 Marker; 1: Positive control; 2: Negative control; 3: Water control; 4: bacterial solution PCR; (**C**) PCR detection of *ThTrx5* gene LR reaction M: DL2000 Marker; 1:Water control; 2: Positive control; 3-5:bacterial solution PCR.

(A) (B)

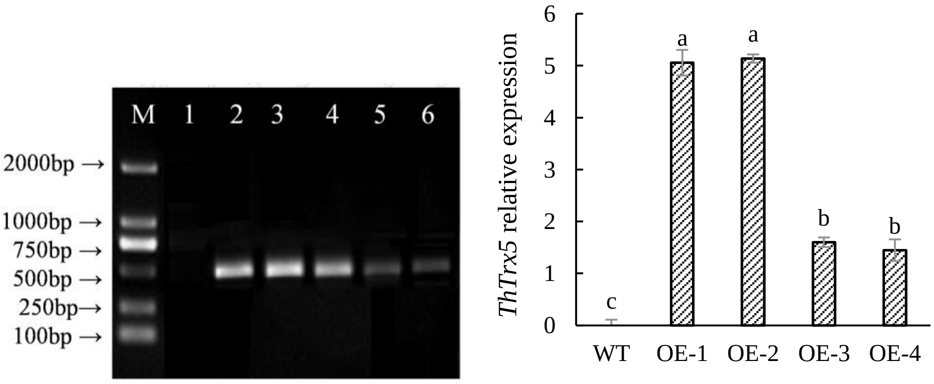

Supplementary Figure S3 Molecular Detection of Transgenic *Arabidopsis* T3 Plants (**A**) M: DNA Maker DL2000;1: water control; 2: positive control; 3-6: transgenic lines; (**B**) *ThTrx5* relative expression detection.

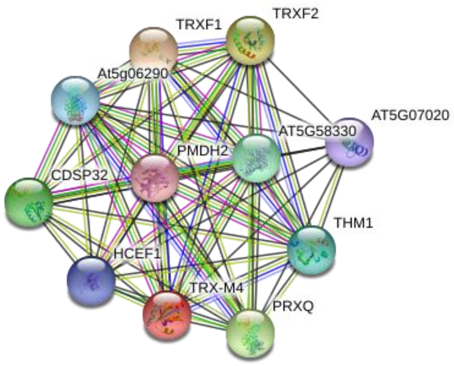

Supplementary Figure 4. Schematic diagram of predicted AtTHM4 interacting proteins.
